# Examining Differences in Pathological Outcomes and Safety for Prostate Cancer Patients Undergoing Either Subcapsular Orchiectomy or Medical Androgen Deprivation Therapy: A Systematic Review

**DOI:** 10.12688/f1000research.161612.2

**Published:** 2025-11-10

**Authors:** Erick Frapancah, Indrawarman Soerohardjo

**Affiliations:** 1 Resident of Urology Division, Department of Surgery, Sardjito Hospital, Faculty of Medicine, Nursing, and Public Health, Gadjah Mada University, Yogyakarta, 55284, Indonesia; 2 Staff of Urology Division, Department of Surgery, Sardjito Hospital, Faculty of Medicine, Nursing, and Public Health, Gadjah Mada University, Yogyakarta, 55284, Indonesia

**Keywords:** Keywords Prostate cancer, Subcapsular orchiectomy, Androgen deprivation therapy, Pathological outcomes, Comparative study, Systematic review

## Abstract

**Purpose:**

The purpose of this systematic review is to investigate differences in pathological outcomes and safety between subcapsular orchiectomy and pharmacological androgen deprivation therapy (ADT) for prostate cancer management.

**Methods:**

A systematic search was conducted on PubMed, Google Scholar, and ScienceDirect for original articles published until February 2024 that compared tumour characteristics, biochemical markers, and adverse effects associated with these treatments. The risk of bias from each study was assessed using the Newcastle Ottawa Scale and Risk of Bias-2 (ROB-2) tool.

**Results:**

Eleven studies meeting the inclusion criteria were analysed. Both subcapsular orchiectomy and pharmacological ADT effectively reduced tumour size and prostate-specific antigen (PSA) levels. Subcapsular orchiectomy was linked to higher surgical complication rates. At the same time, due to its systemic pharmacological mechanisms, pharmacological ADT carries a greater risk of metabolic side effects, such as weight gain and insulin resistance.

**Conclusions:**

Both subcapsular orchiectomy and pharmacological ADT are viable options for prostate cancer treatment. However, their differing safety and pharmacological profiles highlight the need for personalised treatment strategies based on individual patient factors and preferences.

**PROSPERO registration:**

CRD42025634678 (17/01/2025).

## Introduction

Prostate cancer remains a prominent global health concern, affecting millions of men annually and contributing significantly to cancer-related morbidity and mortality. ^1^ Among the principal treatment modalities, androgen deprivation therapy (ADT) is a cornerstone in managing advanced prostate cancer by lowering testosterone levels to inhibit tumour growth and progression. Historically, two primary approaches have been employed to achieve androgen suppression: surgical androgen deprivation, typically via subcapsular orchiectomy, and pharmacological ADT utilising gonadotropin-releasing hormone (GnRH) agonists or antagonists. Although both methods aim to achieve castrate-level testosterone suppression, they differ markedly in invasiveness, reversibility, and patient acceptability. ^2^

Subcapsular orchiectomy, introduced by Riba et al. (1947), involves surgically removing the subcapsular tissue of the testes, effectively halting testosterone production while preserving the external testicular structure. In contrast, pharmacological ADT employs pharmaceutical agents to achieve androgen suppression without surgical intervention. Until the late 1980s, surgical castration was the predominant approach; however, the advent of extended-release GnRH analogues revolutionised the field, offering a non-invasive alternative with psychological and procedural benefits. ^3^ Despite their shared therapeutic goal, emerging evidence suggests that subcapsular orchiectomy and pharmacological ADT may yield distinct pathological outcomes and safety profiles, prompting the need for a systematic evaluation of these differences. ^4^

ADT-induced hypogonadism is associated with several adverse effects, including osteoporosis, an increased risk of fractures, cardiovascular complications, metabolic disturbances, and anaemia. However, there is limited direct comparison of the adverse effect profiles and clinical outcomes between surgical and medical ADT. Existing studies provide inconsistent findings regarding the superiority of one approach over the other in terms of disease control and management of side effects, underscoring the necessity for comprehensive investigation. ^5– 7^

This systematic review aims to evaluate and compare pathological outcomes, including disease progression, survival rates, and adverse effects, in prostate cancer patients undergoing either subcapsular orchiectomy or pharmacological ADT. By synthesising evidence from clinical studies and trials, the review seeks to elucidate the impact of these treatment modalities on long-term outcomes and patient well-being. These findings will provide valuable insights to guide clinicians and patients in selecting treatment strategies that optimise therapeutic efficacy while prioritising quality-of-life considerations. ^8^

## Methods

This systematic review was conducted according to the Preferred Reporting Items for Systematic Reviews and Meta-Analyses (PRISMA) guideline. ^9^ The literature search was performed for evaluate and compare pathological outcomes, including disease progression, survival rates, and adverse effects, in prostate cancer patients undergoing either subcapsular orchiectomy or pharmacological ADT up to February 2024. The protocol of the present systematic review was registered with PROSPERO (CRD42025634678) (date of registration—17/01/2025).

### Study selection

A complete literature search was performed across three electronic databases: PubMed, Science Direct, and Google Scholar. This systematic review included peer-reviewed literature published in the English language. The papers published until February 2024 that met the pre-determined inclusion and exclusion criteria were included in this review.

Inclusion criteria: Examined pathological outcomes, including tumour characteristics, biochemical markers, or adverse effects, in prostate cancer patients treated with subcapsular orchiectomy or medical androgen deprivation therapy (ADT). Directly or indirectly, outcomes between these two treatment methods were compared through randomised controlled trials, cohort studies, case-control studies, and observational studies. Involved adult male patients with a confirmed diagnosis of prostate cancer. Were published in peer-reviewed journals.

Exclusion criteria: Reviews, commentaries, editorials, or conference abstracts without original data. They did not provide adequate information on pathological outcomes relevant to the comparison. They were conducted on non-human subjects. The full text was unavailable.

### Search strategy

The search strategy involved using Medical Subject Headings (MeSH) terms and keywords like “prostate cancer,” “prostate neoplasm,” “androgen deprivation therapy,” and “subcapsular orchiectomy.” The search was limited to English-language articles with no regional restrictions. Additionally, manual searches were done in relevant journals and the reference lists of selected studies to find any articles not indexed in the electronic databases.

### Selection process

The databases were searched by two authors (RIC and IDW) using the search strategy, followed by the removal of duplicates, if any. Title and abstract screening was performed by both authors independently, where each responded with a ‘Yes,’ ‘No,’ or ‘Maybe’ for each entry based on the inclusion and exclusion criteria. Any disagreements were resolved through discussion by mutual consensus. The articles included the following title and abstract screening and underwent independent full-text review by both authors. Disagreements were resolved through discussion with a second author (IDW). The articles that qualified at this stage were finalised for quality appraisal and data extraction for this systematic review.

### Quality assessment

The methodological quality of the included studies was assessed using tools appropriate to their design, such as the Cochrane Risk of Bias Tool for randomised controlled trials and the Newcastle-Ottawa Scale for observational studies. Each study was evaluated for potential biases in selection, performance, detection, attrition, reporting, and other relevant domains.

### Data extraction

Two independent reviewers evaluated the titles and abstracts of identified studies for relevance according to the inclusion and exclusion criteria. Full-text articles of studies deemed potentially relevant were obtained and assessed for eligibility. Disagreements between reviewers regarding study eligibility were resolved through discussion and consensus. Data extraction was conducted using a standardised form, capturing details such as study characteristics (author, publication year, and study design), participant demographics (sample size, age, and allocation), interventions (type of ADT or subcapsular orchiectomy), and outcomes of interest (pathological outcomes and adverse effects).

## Results

**Figure 1.**
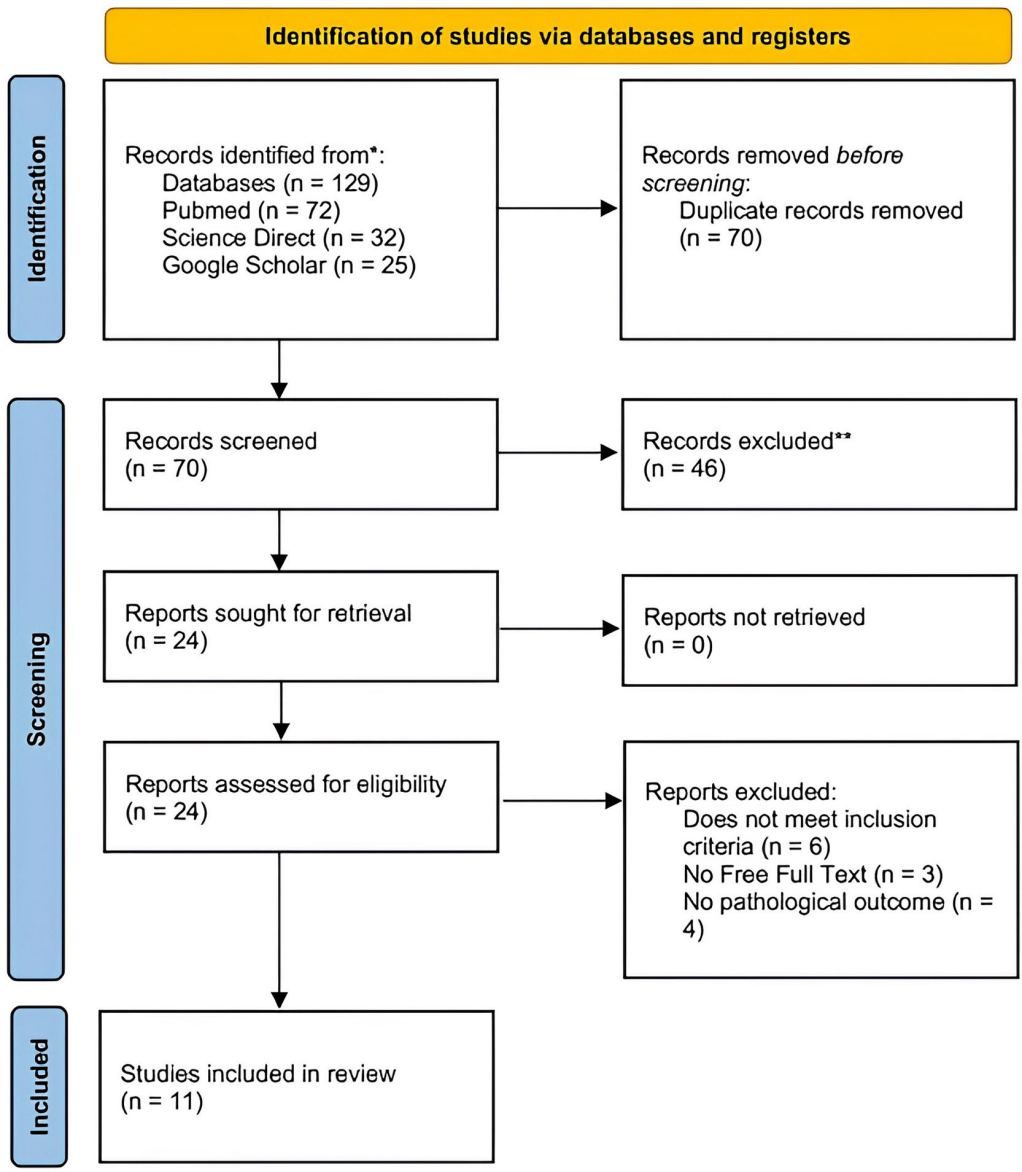
PRISMA flowchart of the included studies. ^9^

The PRISMA flow diagram representing the results of the review process is depicted in Figure 1.

An initial search yielded 129 publications. After removing duplicate entries, 70 records were screened for eligibility. Of these, 46 records were excluded for being unrelated to the topic. Following a detailed evaluation, four records were ineligible articles because they did not meet the inclusion criteria, three were inaccessible in full text, and four did not have pathological outcomes. Ultimately, 11 records met the inclusion criteria and were incorporated into the review that consisted of 7 Randomized Controlled Trials (RCTs), 3 retrospective cohort, and 1 prospective cohort studies ( Figure 1). Summaries of the selected studies are provided in Table 1.

**Table 1.** Eligible studies and summary characteristics.

Study, year	Number of patients	Study type	Nation	Subcapsular orchiectomy	Medical androgen deprivation therapy	Outcome
**Kaisary et al., 1991.**	358	Randomized Clinical Trial	UK	144 patients with a mean age of 72.5 ± 55.89	148 patients with a median age of 71.8 (49-86)	- Both Zoladex and orchiectomy were effective in lowering testosterone levels in metastatic prostate cancer cases. - Both had similar response rates, treatment failure times, and survival outcomes.
**Østergren et al., 2019.**	58	Randomized Clinical Trial	Denmark	28 patients with a mean age of 72 ± 8.8	29 patients with a mean age of 75 ± 5.8	- There is no significant difference in fasting plasma glucose changes between group treated with a gonadotropin-releasing hormone agonist and orchiectomy group in men with advanced prostate cancer.
**Østergren, et al., 2017.**	58	Randomized Clinical Trial	Denmark	28 patients with a mean age of 72 ± 8.8	29 patients with a mean age of 75 ± 5.8	- Triptorelin achieves lower testosterone levels within the castrate range more effectively than orchiectomy in men with prostate cancer.
**Parmar et al., 1987.**	15	Randomized Clinical Trial	UK	7 patients (data for patient's age were not applicable)	8 patients (data for patient's age were not applicable)	- No notable difference is observed in response or disease progression between the D-Trp-6-LHRH and orchiectomy groups. - Long-acting D-Trp-6-LHRH was a safe and effective alternative to orchiectomy for treating advanced prostate cancer.
**Ryan et al., 1998.**	292	Randomized Clinical Trial	UK	144 (data for patients' age were not applicable)	148 (data for patient's age were not applicable)	- Repeated Zoladex treatments lowered testosterone to castrate levels, with similar results in response, duration, treatment failure time, and survival between both groups.
**Soloway et al., 1991.**	162	Randomized Clinical Trial	USA	83 patients with a median age of 70 (46-92)	81 patients with a median age of 68 (48—82)	- Zoladex and orchiectomy were equally effective in treating Stage D2 prostate cancer - Both lowering testosterone to castrate levels within the first month.
**Vogelzang et al., 1995.**	283	Randomized Clinical Trial	USA	145 patients with a median age of 69 (46-92)	138 patients with a median age of 69 (44-95)	- In patients with Stage D2 prostate cancer, goserelin and orchiectomy had similar outcomes - There is no significant effect of treatments or baseline testosterone levels on survival outcomes.
**Garje, et al., 2020.**	33,585	Retrospective cohort	Multiple geographic location	1,328 patients >65 years old and 657 <65 years old	20,847 patients >65 years old and 10,753 <65 years old	- Surgical castration is as effective as medical castration for metastatic prostate cancer, with no difference in overall survival (OS) outcomes.
**Selvi et al., 2019.**	123	Retrospective cohort	Turkey	63 patients with a mean age of 70.22 ± 5.65	60 patients with a mean age of 69.83 ± 5.28	- Subcapsular orchiectomy caused fewer psychosocial side effects compared to total orchiectomy and showed similar outcomes to LHRH analog treatment.
**Tan, et al., 2020.**	523	Retrospective cohort	Singapore	151 with a median age of 73	372 with a median age of 73	- Surgical Orchidectomy (SO) and Medical Castration offer similar results and side effects in treating metastatic prostate cancer (mPCa). - SO is more cost-effective for long-term androgen deprivation therapy (ADT).
**Vargas et al., 2016.**	102	Prospective cohort	Brazil	46 patients with a mean age of 72.4 ± 7.7	56 patients with a mean age of 67.3 ± 6.5	- LHRH analogs caused a bigger drop in hemoglobin levels, worse insulin resistance, more severe anemia, and greater bone loss compared to bilateral orchiectomy, especially in diabetic patients.

Using the Cochrane Risk of Bias tool, four studies were identified as having a low risk of bias, three were classified as having an unclear risk, and one article was determined to have a high risk of selection bias. Based on the Newcastle Ottawa Scale, one study was of moderate quality, and four were of high quality in Tables 2 and 3.

**Table 2.** Newcastle Ottawa Scale Cohort.

Study	Selection				Comparability		Outcome			Total (9/9)
	Representative of the exposed cohort	Selection of external control	Ascertainment of exposure	Outcome of interest not present at the start of the study	Comparability of Cohorts		Assessment of outcomes	Sufficient follow-up time	Adequacy of follow-up	
					Main Factor	Additional Factor				
Garje, et al., 2020	*	*	*	*	*	0	*	*	*	8/9
Selvi et al., 2020	*	0	*	*	*	0	*	*	*	7/9
Tan, et al., 2020	*	*	*	*	*	0	*	*	*	8/9
Vargas et al., 2016	*	*	*	*	*	0	*	*	*	8/9

**Table 3.** ROB-2.

Author	Randomisation process	Deviations from the intended interventions	Missing outcome data	Measurement of the outcome	Selection of the reported result	Overall
** Kaisary et al. 1991**	Low	Low	Low	High	Moderate	Moderate
**Østergren et al. 2019**	Moderate	Moderate	Low	Moderate	Low	Moderate
**Østergren, et al. 2017**	Low	Moderate	Low	Low	Low	Low
**Parmar et al. 1987**	High	Low	Low	High	High	High
**Ryan et al. 1988**	Low	Low	Low	High	Low	Low
**Soloway et al. 1991**	Moderate	Low	Low	Low	High	Low
**Vogelzang et al. 1995**	Low	Moderate	Low	High	High	Moderate

Østergren et al. conducted a comparative study examining the impact of Luteinizing Hormone-Releasing Hormone (LHRH) agonists and subcapsular orchiectomy on testosterone reduction in men with prostate cancer. After adjusting for baseline levels of testosterone, DHEAS, and androstenedione, serum testosterone levels were 29% lower (95% CI 17.2, 41.7) following triptorelin therapy compared to subcapsular orchiectomy (p < .001). Within 48 weeks of follow-up, all patients achieved testosterone levels below 30 ng/dL. The proportion of patients with testosterone levels below 20 ng/dL was 79% for subcapsular orchiectomy and 97% for triptorelin at 12 weeks (p < .05), 92% and 90% at 24 weeks (p = .73), and 87% and 100% at 48 weeks (p < .05), respectively. While LH and FSH levels increased as expected after subcapsular orchiectomy, they were suppressed in the triptorelin group, with LH consistently remaining below the lower reference limit (LRL) except for three patients at the 24-week visit. One of these patients had testosterone levels above the castrate threshold (>50 ng/dL) at that visit, but levels returned to the castrate range by 48 weeks. No significant differences were observed between the two treatment groups regarding their effects on estradiol or adrenal androgens over time. Both groups experienced significant reductions in DHEAS, androstenedione, 17-hydroxyprogesterone, and estradiol levels (p < .001). ^10^

In 2019, Østergren et al. conducted a study to compare the metabolic effects of two androgen deprivation therapy (ADT) methods for advanced prostate cancer: gonadotropin-releasing hormone (GnRH) agonists and orchiectomy. Both treatments showed minimal differences in fasting plasma glucose levels, with a relative change of 0.2 mmol/L at 48 weeks (95% CI -0.1; 0.4), suggesting neither approach significantly affected blood sugar levels. However, the orchiectomy group experienced more pronounced increases in total fat mass, subcutaneous adipose tissue (SAT), and body fat percentage compared to the GnRH agonist group. By 48 weeks, these increases were +2.06 kg, +133 cm ^3^, and +1.3%, respectively, all statistically significant (P < .05). Additionally, the orchiectomy group showed a more significant average weight gain of 3.30 kg compared to the GnRH agonist group (P = .02), primarily attributed to increases in fat mass, especially SAT. A moderate correlation was observed between the rise in fat mass and SAT and the increase in insulin resistance, as measured by HOMA-IR (r = 0.436, P < .001 and r = 0.366, P < .001, respectively). These findings suggest that orchiectomy may pose a higher risk of fat accumulation and insulin resistance compared to GnRH agonists. The study highlights that the choice of ADT can influence metabolic outcomes, with orchiectomy being more strongly associated with increased fat accumulation and a higher potential for insulin resistance. ^11^

Tan et al. conducted a study to assess and compare the outcomes, adverse effects (AEs), and costs of two primary androgen deprivation therapy (ADT) methods for metastatic prostate cancer (mPCa): surgical orchiectomy (SO) and medical castration (MC). The findings offer valuable insights into these treatments’ efficacy, safety, and economic aspects. The study revealed that SO and MC are similarly effective in managing metastatic prostate cancer, as demonstrated by comparable PSA response rates and androgen suppression levels. The PSA response rate (PSA <1 ng/ml) was 65.6% for SO and 67.2% for MC, showing no significant difference in their ability to reduce PSA levels, a key marker of prostate cancer activity. Time to castrate resistance, defined as the period until cancer becomes unresponsive to ADT, was also similar between the two groups (18 months for SO versus 16 months for MC). ^12^

Furthermore, overall survival rates were comparable, with median survival times of 42 months for SO patients and 38.5 months for MC patients, indicating equivalent effectiveness in prolonging life. Significant differences were observed in the adverse effect profiles of SO and MC. Both treatments showed similar outcomes regarding haemoglobin level changes, newly diagnosed diabetes mellitus, coronary artery disease events, and skeletal-related fractures, suggesting comparable safety profiles. One key finding was the cost advantage of SO over MC. After accounting for government subsidies and inflation, the median cost of SO was significantly lower than that of MC (5275 vs. 9185.80), highlighting SO’s greater cost-effectiveness. ^13^ Regarding survivability, Garje et al. reported 5-year overall survival (OS) rates of 24.3% for patients receiving medical castration and 18.3% for those undergoing surgical castration. Although an initial analysis showed a notable survival difference between the groups, this disparity was no longer statistically significant after multivariable adjustments for various factors. This suggests that the choice of castration method may not play a decisive role in determining overall survival. ^13^

A prospective study by Vargas et al. examines notable metabolic changes, including insulin resistance and the onset of diabetes mellitus, in prostate cancer patients undergoing androgen deprivation therapy (ADT). The study revealed that patients treated with LHRH analogues experienced worse insulin resistance outcomes than those undergoing bilateral orchiectomy. Specifically, the LHRH analogue group demonstrated higher fasting glucose levels, basal insulin levels, and HOMA-IR (Homeostatic Model Assessment for Insulin Resistance) scores, indicating an elevated risk of diabetes. Anaemia was also observed, with patients on LHRH analogues showing a more significant reduction in haemoglobin levels than those who underwent surgical castration, suggesting a more substantial impact of LHRH analogues on erythropoiesis (red blood cell production). Furthermore, bone health was negatively affected across all ADT recipients, with LHRH analog-treated patients exhibiting more pronounced bone demineralisation, particularly in the lumbar spine. The study reported a significantly more significant loss of bone mineral density (BMD) in the lumbar spine for the LHRH analogue group than those with bilateral orchiectomy. ^5^

A randomised clinical trial conducted by Soloway et al. revealed that the effectiveness of the two treatments was highly comparable. An objective response—complete response, partial response, or stable disease—was achieved in 81% of patients treated with Zoladex and 78% of those undergoing orchiectomy. This difference was not statistically significant, indicating similar efficacy between the treatments. Additionally, the time to treatment failure, primarily attributed to disease progression, showed no significant variation between the Zoladex and orchiectomy groups, further supporting their comparable effectiveness over time. ^14^

Selvi et al. compared the effects of total orchiectomy, subcapsular orchiectomy, and LHRH analogue therapy in patients with hormone-sensitive metastatic prostate cancer. The study concluded that all three treatments effectively achieved therapeutic castration levels, successfully reducing testosterone to the desired range. Patients undergoing subcapsular orchiectomy reported higher satisfaction levels than those undergoing total orchiectomy, suggesting that subcapsular orchiectomy, being less invasive, may be more favourable due to its organ-preserving nature and perceived less radical approach. Health-related quality of life (HRQoL) scores were also higher in the subcapsular orchiectomy group, likely due to the reduced psychological impact of this organ-preserving method. Furthermore, the frequency and severity of post-traumatic stress (PTS) and post-traumatic stress disorder (PTSD) were lower in patients who underwent subcapsular orchiectomy compared to those who had total orchiectomy. Patient satisfaction in the subcapsular orchiectomy group was comparable to those receiving LHRH analogue therapy. ^15^

Research conducted by Kaisary et al. compared the efficacy of the LHRH analogue Zoladex with orchiectomy in managing metastatic prostatic carcinoma. The study found that both Zoladex and orchiectomy were equally effective in reducing serum testosterone levels to the surgically castrate range, demonstrating their success in achieving the necessary hormonal suppression for treating the condition. The time between the two groups was comparable until treatment failure or patient withdrawal. Additionally, at a median follow-up of two years, no significant difference in overall survival was observed between patients receiving Zoladex and those undergoing orchiectomy. ^16^

A study by Agarwala et al. provides an in-depth comparison of the outcomes between surgical and medical castration for treating metastatic hormone-sensitive prostate cancer (mHSPC). The analysis revealed no significant difference in the time to progression (TTP) between patients undergoing bilateral orchiectomy and those receiving medical castration. The mean TTP was 13.9 months for the bilateral orchiectomy group and 13.8 months for the medical castration group, indicating that both methods are equally effective in delaying the progression of mHSPC to castration-resistant prostate cancer. The mean nadir PSA levels were lower in the surgical group (4.7 ng/ml) compared to the medical group (9.8 ng/ml). However, the time required to reach the nadir PSA level was nearly identical, averaging 8.7 months in the surgical group and 8.8 months in the medical group. This suggests that while both treatments successfully lower PSA levels, surgical castration may achieve a more significant reduction. Additionally, testosterone suppression was more pronounced in the surgical group during the follow-up period, though the difference was not statistically significant. ^17^

Vogelzang et al. conducted a study comparing the efficacy and safety of goserelin, a luteinizing hormone-releasing hormone (LHRH) agonist, with orchiectomy (surgical removal of the testes) in patients with Stage D2 prostate cancer. By the fourth week, serum testosterone levels had decreased to castrate levels in both groups and remained stable throughout the treatment. Reductions in acid and alkaline phosphatase levels were also observed, indicating a positive response to treatment. The objective response rates were comparable between the groups, 82% for goserelin and 77% for orchiectomy, with no statistically significant differences after adjusting for stratification factors. Median time to treatment failure was nearly the same for both treatments, and survival times showed no significant differences, demonstrating similar effectiveness. Both treatments were well tolerated, with the most common adverse events reported being pain, hot flashes, and lower urinary tract symptoms. The pattern of adverse events was consistent across both groups, with no significant differences noted. ^18^

A Randomized Controlled Trial conducted by Parmar et al. in 1985 sought to evaluate and compare the safety and efficacy of two treatments for advanced prostate carcinoma: a slow-release formulation of D-Trp-6 luteinizing hormone-releasing hormone (D-Trp-6-LHRH) microcapsules and orchiectomy. Both treatment approaches effectively suppressed testosterone levels and reduced prostatic acid phosphatase levels, demonstrating equivalent effectiveness in disease management. A treatment response, either improvement or stabilization, was observed in 87% of patients treated with D-Trp-6-LHRH microcapsules and 81% of those who underwent orchiectomy, highlighting comparable efficacy between the two methods. Testosterone suppression-related side effects were similar in both groups, suggesting that the safety profile of D-Trp-6-LHRH microcapsules aligns with that of orchiectomy. Follow-up evaluations revealed a slight trend toward reduced psychological distress in patients receiving hormonal treatment, although no statistically significant differences in psychological outcomes were detected between the groups. ^19^

In 1987, Parmar et al. conducted a study revealing that the psychological distress linked to orchiectomy, the surgical removal of one or both testicles, was only marginally more significant than that experienced by patients receiving alternative treatments. Both orchiectomy and D-Trp-6-LHRH therapy were shown to be effective in managing advanced prostate cancer. Orchiectomy provides the benefit of an immediate reduction in testosterone levels without requiring continuous medication. However, it is associated with the risks inherent to surgical procedures and anaesthesia, and some patients may not respond to this treatment. ^20^

Ryan et al. conducted a detailed study comparing the efficacy of the LH-RH analogue Zoladex and orchidectomy in managing metastatic prostate cancer. The findings demonstrated that administering Zoladex every 28 days was equally effective as orchidectomy in reducing serum testosterone levels to the castrate range. Both treatment approaches yielded comparable subjective and objective response rates. Furthermore, the two groups had no significant differences in the duration of response, time to treatment failure, or survival rates. Follow-up data collected ten months after trial recruitment concluded that Zoladex and orchidectomy are equally effective options for treating metastatic prostate cancer. ^21^

## Discussion

The ongoing debate between surgical and chemical methods for achieving castration-level testosterone in prostate cancer treatment highlights differing preferences. Many patients tend to favour pharmacological approaches over surgery due to their non-invasive nature, avoidance of permanent physical changes, and potentially reduced risk of immediate postoperative complications. ^22, 23^ Reducing testosterone levels plays a vital role in managing prostate cancer by slowing disease progression and potentially enhancing patient survival. ^24, 25^ A study suggests that LHRH agonist therapy is more effective in achieving testosterone reduction to the target castrate range. ^16, 20^ LHRH agonists ensure sustained testosterone suppression over time. With an appropriate dosing schedule, patients can consistently maintain castrate testosterone levels through regular injections. In contrast, surgical outcomes may differ based on the method and thoroughness of the orchiectomy. ^26^ In contrast to subcapsular orchiectomy, a surgical intervention, LHRH agonists are delivered via injections. This non-invasive approach minimizes the risk of surgical complications, including postoperative scrotal swelling or hematoma, which some patients experience after orchiectomy. Additionally, pharmacological treatment provides the benefit of being reversible. ^27^ If side effects become unmanageable or a patient decides to discontinue treatment, ceasing LHRH agonist therapy allows testosterone levels to gradually return to their normal range. ^28^

Subcapsular orchiectomy achieves androgen deprivation by surgically removing the testes, which serve as the primary source of testosterone production. Testosterone plays a critical role in driving prostate cancer progression and PSA production. By eliminating testosterone production, this surgical approach effectively lowers PSA levels. Similarly, medical androgen deprivation therapy (ADT) achieves over 95% suppression of androgen levels, reducing testosterone to below 50 ng/dL. This substantial decrease in testosterone also results in a significant reduction in PSA levels. ^4, 17^

The findings of this review demonstrate that both subcapsular orchiectomy and medical ADT effectively reduce prostate-specific antigen (PSA) levels. These results align with the systematic review by Zhang et al., which reported similar outcomes between these treatment modalities. However, it is essential to acknowledge that the high heterogeneity among studies included in this review influences the ability to draw definitive conclusions. Some studies prioritized oncological parameters, such as testosterone and PSA levels, while others examined progression, metabolic effects, psychological impacts, or survival outcomes associated with these treatments. ^13, 14, 16, 18– 20^

A recent study by Osunaiye et al. observed a significant correlation between the percentage changes in serum testosterone and PSA levels two months after orchidectomy (P < 0.001). However, no significant correlation was found at four and six months, suggesting the possibility of subclinical tumour progression despite achieving the castrated state. These findings highlight the importance of serum PSA as a key biomarker for monitoring prostate cancer progression and treatment effectiveness. The extent and duration of PSA decline following orchidectomy appear to vary, influenced by the surgical approach and underlying tumour dynamics. ^11^ PSA is a highly sensitive marker for predicting prostate cancer in symptomatic patients, with an estimated sensitivity of around 93%. However, its specificity is relatively low, at approximately 20%. When combined, the testosterone-to-PSA ratio enhances diagnostic accuracy, achieving a sensitivity of 79.17% and a specificity of 89.01%. ^29^

Progression, defined as two consecutive increases in total PSA following the achievement of a nadir PSA or the development of new or worsening lesions on bone scans or soft tissue lesions (based on RECIST criteria), showed no significant differences between subcapsular orchiectomy and medical ADT. ^14, 17, 19^ This phenomenon can be attributed to the shared objective and mechanism of action of both modalities, as androgen suppression drives the progression control of prostate cancer in both approaches. However, factors like race, income level, and the type of healthcare facility may influence the treatment choice and potentially impact progression outcomes. ^17^

ADT therapy has been linked to heightened insulin resistance, a central feature of metabolic syndrome. This increased resistance can result in elevated fasting glucose levels and a greater likelihood of developing diabetes mellitus. ^10, 30^ Patients undergoing ADT have been observed to experience unfavourable alterations in lipid profiles, such as increased levels of total cholesterol, LDL cholesterol, HDL cholesterol, and triglycerides. ^30^ These changes elevate the risk of cardiovascular diseases. ^1, 10^ Additionally, one study indicated that neither treatment significantly affected blood sugar levels. ^31^ This variability may result from compensatory mechanisms that remain unaffected by androgen levels. ^32^

ADT has been associated with adverse effects on psychological well-being. ^15, 20^ Research indicates that patients undergoing orchiectomy may experience more significant psychological distress compared to those receiving medical castration with LHRH analogues. This is primarily attributed to the irreversible nature of the surgery and its immediate physical and emotional consequences. Orchiectomy often leads to decreased libido and impotence, which can significantly impact a patient’s self-esteem and intimate relationships. The resulting loss of sexual function may provoke feelings of inadequacy and depression. Additionally, physical changes such as the removal of the testes and alterations in body composition can contribute to body image concerns. ^30^ Many patients report feeling less masculine or incomplete, exacerbating their psychological distress. ^15^ Some individuals also experience phantom testis syndrome, characterized by sensations or pain in the area where the testes were removed, serving as a persistent reminder of the surgery and further contributing to psychological discomfort. ^15, 18^

Most studies concluded no significant differences between the two modalities regarding survivability. ^13, 14, 16, 18^ At present, no definitive explanation exists for this outcome. However, risk factor adjustments or sample selection variations could influence survivability results, as patients with differing risk stratifications may experience varying outcomes. Potential confounding biases and influencing factors should be carefully considered. ^31, 33^

This systematic review has certain limitations. As mentioned earlier, the substantial heterogeneity among studies complicates the ability to draw definitive conclusions. Furthermore, advancements in medical technologies may have introduced variability, as differences in surgical techniques and medical ADT drugs utilised across studies conducted in different years could significantly influence outcomes, increasing the potential for bias. Consequently, carefully designed future studies are strongly recommended to produce more reliable and precise results.

### Future direction

Further research is needed to evaluate the long-term comparison between subcapsular orchiectomy and medical androgen deprivation therapy (ADT) in managing prostate cancer. Future studies should focus on the psychological, metabolic, and oncological impacts of both methods, as well as their potential side effects. Additional research is also required to examine the effects of ADT on insulin resistance and cardiovascular risk, as well as the psychological impact of physical changes due to orchiectomy. More well-designed, controlled intervention studies will provide clearer insights for better patient management.

## Conclusion

This systematic review underscores the similar effectiveness of subcapsular orchiectomy and medical ADT in producing favourable pathological outcomes for prostate cancer patients. However, selecting between these treatment options should account for therapeutic effectiveness, patient-specific considerations, and potential side effects. Future prospective studies with standardised protocols are needed to determine the most suitable androgen deprivation therapy approach for prostate cancer patients.

## Data Availability

No data are associated with this article. Figshare: Examining Differences in Pathological Outcomes and Safety for Prostate Cancer Patients Undergoing Either Subcapsular Orchiectomy or Medical Androgen Deprivation Therapy: A Systematic Review. http://doi.org/10.6084/m9.figshare.28300943. ^32^
• This project contains the below mentioned reporting guidelines. Prisma diagram.jpg • Prisma 2020 Checklist.docx Data are available under the terms of the Creative Commons Attribution 4.0 International license (CC-BY 4.0).
