# Supplementary material for: Examining Differences in Pathological Outcomes and Safety for Prostate Cancer Patients Undergoing Either Subcapsular Orchiectomy or Medical Androgen Deprivation Therapy: A Systematic Review
Source: F1000Res. 2025 Nov 10;14:254. Originally published 2025 Mar 3. [Version 2] doi: 10.12688/f1000research.161612.2 (PMC12504932; doi:10.12688/f1000research.161612.2)
Supplement: Peer review history

# Reviewer report: Reviewer response for version 2

**DOI:** 10.5256/f1000research.189352.r431087

**Published:** 2025-11-13

**Authors:** Syah Mirsya Warli

**Affiliations:** 1 Universitas Sumatera Utara, Medan, Indonesia

I have reviewed the revised manuscript and have no further comments from my side. Thank you for the opportunity to review.

Are the rationale for, and objectives of, the Systematic Review clearly stated?

Yes

Is the statistical analysis and its interpretation appropriate?

Yes

If this is a Living Systematic Review, is the ‘living’ method appropriate and is the search schedule clearly defined and justified? (‘Living Systematic Review’ or a variation of this term should be included in the title.)

Not applicable

Are sufficient details of the methods and analysis provided to allow replication by others?

Partly

Are the conclusions drawn adequately supported by the results presented in the review?

Yes

Reviewer Expertise:

Uro-Oncology

I confirm that I have read this submission and believe that I have an appropriate level of expertise to confirm that it is of an acceptable scientific standard.

# Reviewer report: Reviewer response for version 1

**DOI:** 10.5256/f1000research.177664.r412958

**Published:** 2025-10-07

**Authors:** Fakhri Taher

**Affiliations:** 1 University of Indonesia Center for Clinical Epidemiology and Evidence Based Medicine, Jakarta, Special Capital Region of Jakarta, Indonesia

This study aims to summarise the outcomes and adverse effects of orchidectomy and hormonal therapy for prostate cancer. Although these treatment modalities have been established for quite some time, the topic remains relevant in the current clinical landscape.

 The authors have clearly stated the objectives of the study and have provided a general overview of the methodology. However, I would like to request clarification on several points in the **Methods** section:

- Please elaborate on the definition and criteria used for *pathological outcomes*. compared across studies.
- You mentioned that disagreements between authors were resolved by a *third author*. Since only two authors are listed, kindly clarify this process or correct the statement if necessary.
- In the methodology, you stated that literature was searched through electronic databases. However, in the results section, you mentioned an additional publication identified through *other sources*. Could you please clarify what those sources were (manual search, snowballing, or reference checking)?

The authors have done a commendable job in describing the included studies. However, as multiple outcomes are reported across the studies, it would significantly improve clarity if this information were summarised in a **table forma**t. This would allow readers to more easily compare results and draw conclusions.

Are the rationale for, and objectives of, the Systematic Review clearly stated?

Yes

Is the statistical analysis and its interpretation appropriate?

Not applicable

If this is a Living Systematic Review, is the ‘living’ method appropriate and is the search schedule clearly defined and justified? (‘Living Systematic Review’ or a variation of this term should be included in the title.)

No

Are sufficient details of the methods and analysis provided to allow replication by others?

Partly

Are the conclusions drawn adequately supported by the results presented in the review?

Yes

Reviewer Expertise:

Urology Oncology

I confirm that I have read this submission and believe that I have an appropriate level of expertise to confirm that it is of an acceptable scientific standard, however I have significant reservations, as outlined above.

# Response

**Published:** 2025-11-03

**Authors:** Erick Frapancah

**Affiliations:** Urology, Universitas Gadjah Mada, Yogyakarta, Special Region of Yogyakarta, Indonesia

Dear reviewer,

 We sincerely thank you for your constructive and insightful comments on our manuscript. We have carefully revised the paper in accordance with your suggestions. Each point has been addressed in detail below, and the corresponding changes have been incorporated into the revised version of the manuscript:

1. We acknowledge that our operational definition of “pathological outcomes” in the sentence “Examined pathological outcomes, including tumour characteristics, biochemical markers, or adverse effects, in prostate cancer patients treated with subcapsular orchiectomy or medical androgen deprivation therapy (ADT)” within the Study selection section may not have clearly explained the definition used in this study. We have therefore revised the sentence to read: “Examined pathological outcomes including: (1) tumour characteristics (tumour stage [localized vs. metastatic], castration-resistant progression, and time to treatment failure); (2) biochemical markers (serum testosterone level and prostate-specific antigen (PSA) reduction or nadir level); and (3) adverse effects (metabolic changes such as insulin resistance, body mass index, lipid profile; haematologic effects such as anaemia or haemoglobin change; and surgical or psychological complications), in prostate cancer patients treated with subcapsular orchiectomy or medical androgen deprivation therapy (ADT).”
2. We appreciate the reviewer’s observation and agree that this statement required clarification. The text has now been corrected. The screening and data extraction were performed independently by two authors (RIC and IDW). Any disagreements were resolved through discussion and consensus between these two authors; no third author was involved in these processes.
3. We also agree that clarification was required regarding the additional publication mentioned in the Results section. This publication was identified through manual reference checking (snowballing) from the bibliographies of previously selected articles. However, we did not identify any eligible studies that were not indexed within the electronic databases included in our search strategy.

Finally, we have carefully reviewed the entire manuscript to ensure that the terminology, methodology, and descriptions are clear and consistent. We sincerely appreciate the reviewer’s valuable feedback, which has significantly improved the precision, transparency, and readability of our paper. We believe that the revised version now fully addresses all reviewer comments and strengthens the overall scientific quality of the manuscript.

 Respectfully,

 Erick Frapancah

 Indrawarman Soerohardjo (Corresponding Author)

 Division of Urology, Department of Surgery

 Faculty of Medicine, Public Health, and Nursing

 Universitas Gadjah Mada-Dr. Sardjito Hospital, Yogyakarta, Indonesia

# Reviewer report: Reviewer response for version 1

**DOI:** 10.5256/f1000research.177664.r412960

**Published:** 2025-09-23

**Authors:** Syah Mirsya Warli

**Affiliations:** 1 Universitas Sumatera Utara, Medan, Indonesia

Firstly, I would like to commend this manuscript. It is written in accordance with a well-structured PRISMA approach and includes an appropriate PROSPERO registration. The authors have developed a robust search strategy involving multiple databases (PubMed, ScienceDirect, Google Scholar) and the use of appropriate MeSH terms. The application of the Newcastle-Ottawa Scale and ROB-2 tools to different study designs demonstrates the study's rigorous quality assessment. The inclusion and exclusion criteria are sound, with clear definitions for both patient population and outcomes.

 The manuscript effectively emphasizes its clinical relevance by addressing a genuine clinical dilemma with practical implications, covering a comprehensive range of outcomes, including biochemical, metabolic, psychological, and survival measures.

 However, I have several concerns that need to be addressed by the authors. First, regarding the methodology: it was correctly identified that the studies by Arogundade et al. and Islam et al. do not provide direct comparisons between surgical and medical ADT, yet they were included. This represents a fundamental violation of the stated inclusion criteria and compromises the validity of the conclusions. I suggest either excluding these studies or modifying the inclusion criteria and providing a clear justification for the inclusion of single-arm studies. If these studies are retained, I encourage the authors to perform sensitivity analyses that exclude them.

 My second concern relates to incomplete search documentation. The phrase “The literature search started in [insert start date]” is unprofessional and suggests inadequate manuscript preparation. This must be corrected with the actual search dates. Although this may sound trivial, in my opinion, it is a major flaw.

 Heterogeneity is also an issue. While you acknowledge heterogeneity, the clinical and methodological diversity is substantial—different surgical techniques (subcapsular vs. total orchiectomy), various medical ADT agents with different mechanisms, wide ranges of follow-up periods (months to years), as well as different outcome definitions and measurement timepoints. I suggest providing more detailed subgroup analyses and considering forest plots showing individual study effects even in the absence of meta-analysis.

 Lastly, I want to emphasize some minor issues, such as inconsistencies in citation style, the low image quality of the PRISMA flowchart, and the incorrect usage of a few statistical terms.

 In summary, while this manuscript addresses an important clinical question, employs the correct methodology, and features a comprehensive outcome assessment, there are major flaws that need to be corrected. I encourage the authors to undertake a major revision of the manuscript to meet the standards of a highly esteemed journal.

Are the rationale for, and objectives of, the Systematic Review clearly stated?

Yes

Is the statistical analysis and its interpretation appropriate?

Yes

If this is a Living Systematic Review, is the ‘living’ method appropriate and is the search schedule clearly defined and justified? (‘Living Systematic Review’ or a variation of this term should be included in the title.)

Not applicable

Are sufficient details of the methods and analysis provided to allow replication by others?

Partly

Are the conclusions drawn adequately supported by the results presented in the review?

Yes

Reviewer Expertise:

Uro-Oncology

I confirm that I have read this submission and believe that I have an appropriate level of expertise to confirm that it is of an acceptable scientific standard, however I have significant reservations, as outlined above.

# Response

**Published:** 2025-10-05

**Authors:** Erick Frapancah

**Affiliations:** Urology, Universitas Gadjah Mada, Yogyakarta, Special Region of Yogyakarta, Indonesia

Dear Reviewer,

 Thank you very much for your valuable and insightful comments on our manuscript. Your feedback has been instrumental in helping us improve the quality of our paper. We have addressed all the points raised as follows:

**1. Study inclusion (Arogundade et al. and Islam et al.)**

 We acknowledge that the studies by Arogundade et al. and Islam et al. did not provide direct comparisons between surgical and medical ADT. We recognize that their inclusion was inconsistent with our strict inclusion criteria. Therefore, we have excluded these studies from our analysis and include them as “studies not meeting the inclusion criteria” in the PRISMA flowchart (Screening section). After reanalysis, we confirmed that the exclusion of these studies did not affect the overall results or conclusions of our systematic review.

**2. Literature search clarification**

 We also acknowledge the oversight in proofreading that resulted in the placeholder phrase “The literature search started in [insert start date]”. This has been corrected, and we confirm that our literature search included all eligible studies published up to February 2024.

**3. High Heterogeneity**

 We summarized the key findings from each included study in a structured manner, focusing on trends in oncologic outcomes, survival, and adverse effects between surgical orchiectomy and medical ADT. We qualitatively explored potential sources of heterogeneity, including differences in study design, treatment duration, and follow-up period, to better understand variations in results across studies. The methodological quality of each study was assessed using standardized criteria to support a more reliable interpretation of the evidence. Finally, we discussed both the consistency and discrepancies among the studies to highlight areas where findings converged or differed, thereby providing a comprehensive narrative synthesis of the current evidence.

**4. Minor revisions and formatting improvements**

 We have addressed all minor issues, including inconsistencies in citation style and figure quality. The PRISMA flowchart has been updated with higher resolution, and statistical terminology has been refined for clarity.

 All deleted text is shown with a strikethrough and yellow highlight, while all revised or added text is highlighted in red. Following the exclusion of the two aforementioned studies, we also updated the reference numbering accordingly.

 We sincerely hope that these revisions improve the quality of our manuscript and that it now meets the publication standards of F1000Research.

 Thank you once again for your constructive comments and valuable time.

 Warm regards,

 Erick Frapancah

 (on behalf of all authors)

 Urology Division, Department of Surgery, Sardjito Hospital, Faculty of Medicine, Nursing, and Public Health, Gadjah Mada University, Yogyakarta, 55284, Indonesia

# Reviewer report: Reviewer response for version 1

**DOI:** 10.5256/f1000research.177664.r397793

**Published:** 2025-09-03

**Authors:** Chaidir Arif Mochtar

**Affiliations:** 1 Universitas Indonesia, Depok, West Java, Indonesia; 2 Urology, Ciptomangunkusumo Hospital - Universitas Indonesia, Central Jakarta, DKI Jakarta, Indonesia

The objectives of this study are clearly described, and the research methods are presented in sufficient detail. In the Methods section, the sentence *"The literature search started in [insert start date]"* appears incomplete. I recommend that the authors clarify whether the initial year of the search is missing or if this is simply a typographical error.

 In addition, while the inclusion criteria specify that eligible studies should compare surgical orchiectomy with ADT, two included studies (Arogundade et al. and Islam et al.) did not provide such a comparison but only reported outcomes of surgical orchiectomy. I suggest the authors address this inconsistency for greater clarity. 

 It should be noted that the studies included in this review were retrospective cohorts rather than prospective ones.

 The statistical approach chosen by the authors is appropriate. Given the heterogeneity of the included studies, the decision not to perform a meta-analysis is justified and well explained. The narrative synthesis is clearly presented.

Are the rationale for, and objectives of, the Systematic Review clearly stated?

Yes

Is the statistical analysis and its interpretation appropriate?

Not applicable

If this is a Living Systematic Review, is the ‘living’ method appropriate and is the search schedule clearly defined and justified? (‘Living Systematic Review’ or a variation of this term should be included in the title.)

Not applicable

Are sufficient details of the methods and analysis provided to allow replication by others?

Yes

Are the conclusions drawn adequately supported by the results presented in the review?

Yes

Reviewer Expertise:

Urologic Oncology

I confirm that I have read this submission and believe that I have an appropriate level of expertise to confirm that it is of an acceptable scientific standard.

# Response

**Published:** 2025-10-05

**Authors:** Erick Frapancah

**Affiliations:** Urology, Universitas Gadjah Mada, Yogyakarta, Special Region of Yogyakarta, Indonesia

Dear Reviewer,

 Thank you very much for your thoughtful and constructive comments on our manuscript. We highly appreciate your detailed review, which has helped us improve the clarity and quality of our work. Our responses to your points are as follows:

**1.Comment 1:**

 “In the Methods section, the sentence ‘The literature search started in [insert start date]’ appears incomplete. I recommend that the authors clarify whether the initial year of the search is missing or if this is simply a typographical error.”

**Response: **We appreciate the reviewer’s observation. This was indeed a typographical oversight that occurred during proofreading. The correct statement now reads: “The literature search was performed for evaluate and compare pathological outcomes, including disease progression, survival rates, and adverse effects, in prostate cancer patients undergoing either subcapsular orchiectomy or pharmacological ADT up to February 2024.” We have revised the text accordingly in the Methods section.

**2.Comment 2:**

 “While the inclusion criteria specify that eligible studies should compare surgical orchiectomy with ADT, two included studies (Arogundade et al. and Islam et al.) did not provide such a comparison but only reported outcomes of surgical orchiectomy. I suggest the authors address this inconsistency for greater clarity.”

**Response: **We thank the reviewer for highlighting this important point. Upon re-evaluation, we acknowledge that the studies by Arogundade et al. and Islam et al. did not meet our strict inclusion criteria, as they did not directly compare surgical orchiectomy with medical ADT. We have therefore excluded these two studies and categorized them as “studies not meeting the inclusion criteria” in the updated PRISMA flowchart (Screening section). We also rechecked the data and confirmed that their exclusion did not affect the results or conclusions of our review.

**3.Comment 3:**

 “It should be noted that the studies included in this review were retrospective cohorts rather than prospective ones.”

**Response: **We thank the reviewer for this clarification. We have updated the manuscript and included 7 Randomized Controlled Trials, 3 Retrospective Cohort, and 1 Prospective cohort studies. This information is now clearly stated in the Results section.

**4. Comment 4:**

 “The statistical approach chosen by the authors is appropriate. Given the heterogeneity of the included studies, the decision not to perform a meta-analysis is justified and well explained. The narrative synthesis is clearly presented.”

**Response: **We sincerely appreciate the reviewer’s positive feedback regarding our methodological approach and presentation.

**5. Additional Revisions:**

**Response: **We have also corrected minor formatting inconsistencies, improved figure quality (including the PRISMA flowchart), and refined statistical terminology for clarity. All deleted text is shown with strikethrough and yellow highlight, while revisions and additions are highlighted in red. Reference numbering has been updated accordingly after the exclusion of the two studies.

 We hope these revisions address all comments satisfactorily and enhance the quality and clarity of our manuscript. Thank you again for your valuable time and insightful feedback.
